# Fathers’ involvement in child care activities: Qualitative findings from the highlands of Madagascar

**DOI:** 10.1371/journal.pone.0247112

**Published:** 2021-03-30

**Authors:** Hasina Rakotomanana, Christine N. Walters, Joel J. Komakech, Deana Hildebrand, Gail E. Gates, David G. Thomas, Fanjaniaina Fawbush, Barbara J. Stoecker

**Affiliations:** 1 Department of Nutritional Sciences, Oklahoma State University, Stillwater, Oklahoma, United States of America; 2 Department of Psychology, Oklahoma State University, Stillwater, Oklahoma, United States of America; 3 Department of Agricultural and Food Science and Technology, University of Antananarivo, Antananarivo, Madagascar; Universitat Luzern, SWITZERLAND

## Abstract

Few studies have investigated fathers’ roles in child care in Madagascar. This study explored the perceptions, attitudes, and practices regarding fathers’ involvement in child care using qualitative methods. Ten focus group discussions were conducted among parents of children aged 6–23 months; seven were among mothers, and three among fathers. In-depth semi-structured interviews (n = 8) were also conducted with key informants. Discussions and interviews were audio-recorded and the verbatim transcripts in Malagasy were translated into English. Data were analyzed using the thematic analysis approach. Provision of financial and material support as well as teaching and playing with the child were the main perceived roles of fathers. In practice, fathers spent their time alone with their children playing and holding them when the mother was unavailable. Busy schedules and separation due to work were major barriers to fathers’ involvement. Traditional gender roles for child care in which the mother is seen as primarily responsible for the child were salient across the data. Consequently, men involved in child care activities and their wives were often criticized by the community. Nevertheless, there was self-reported interest from both mothers and fathers in involving men more in child care. Interventions aimed to increase fathers’ involvement in child care may be more successful when they focus on shifting the community perceptions on the division of responsibilities between fathers and mothers.

## 1. Introduction

The first two years of life offer a unique window of opportunity for a child’s physical growth and development [1–3]. Appropriate child care practices, such as food preparation and feeding, psychosocial stimulation, hygiene practices, and care during illness [3–5] are critical during that period to prevent undernutrition and impaired development. Evidence from sub-Saharan African countries indicates that such activities are most often performed by the mothers as they are regarded as the primary care providers [6–8].

Fathers’ perceived roles typically include the provision of financial and logistical resources for the family [6]. Consequently, most interventions aiming to improve feeding practices and to promote child development target mothers. However, there is emerging evidence on the positive outcomes for child nutrition and development of expanding fathers’ involvement beyond their traditional roles in low- and middle-income countries (LMICs). Nutrition education sessions targeted to fathers improved child feeding practices in Kenya, Ethiopia, and Vietnam [9–11]. Benefits of paternal involvement on child development outcomes have been well documented, although most of the studies were conducted in high-income countries. Children whose fathers were more consistently engaged had higher cognitive scores [12–16], better emotional regulation [13], and improved language skills [17,18]. Engaging fathers in child care activities also can alleviate the workload of mothers, which is in line with the United Nations’ Sustainable Development Goal 5: achieve gender equality and empower all women and girls [19].

Presumably, increasing involvement of fathers in child care has the potential to contribute to the efforts of reducing child undernutrition and promoting child development in LMICs including Madagascar. The country’s high level of poverty [20] combined with the very high child stunting rates (49%) [21] dramatically increase the risk of poor development of almost half of Malagasy children [3,22]. However, designing and implementing such interventions requires a strong understanding of the local context and culture. In Madagascar, parental perceptions of fathers’ roles and participation in child care are not well understood. Thus, this study explored Malagasy parental perceptions, attitudes, and practices regarding fathers’ involvement in child care using a qualitative approach. The main research questions were: 1) what are perceptions of fathers and of the community of fathers’ responsibilities in child care? 2) how are fathers engaged in child care? 3) what are the barriers to fathers’ involvement? 4) what are the perceptions of the community regarding involved fathers? The results of this study provide valuable insights in designing future programs and interventions aiming to increase fathers’ engagement in child care in Madagascar. In this study, father involvement was defined as paternal direct interactions with the child (feeding, holding, playing, bathing) as well as indirect responsibilities related to child care such as providing and preparing food for the child [23].

## 2. Methods

The consolidated criteria for reporting qualitative research (COREQ) guidelines were used to report the methods and the results of this study [24].

### Study participants

This qualitative study was conducted in two rural districts, Antanifotsy and Antsirabe II, of the Vakinankaratra region of Madagascar in April 2019. The Vakinankaratra region, located at 170 km south of the capital city Antananarivo, is one of the most agriculturally productive regions in the country. Paradoxically, the Vakinankaratra region had the highest stunting rate in Madagascar in 2018 (60%) [25]. Seven focus group discussions (FGDs) were conducted among mothers and three FGDs among fathers of children 6–23 months of age. FGDs had an average of 8 to 10 participants and were conducted in the local community nutrition centers. A total of 46 mothers and 17 fathers participated in the FDGs. Purposive sampling was used to select fokontanys (smallest administrative unit) with experienced local health community workers (community nutrition agents or CNAs). The CNAs identified participants who would be willing to speak and contribute to discussions regarding child care and fathers’ involvement. When FGDs were conducted for both mothers and fathers in the same fokontany, participants were selected from different households to avoid conflict and potential bias. Individual in-depth semi-structured interviews (IDI) were conducted with key informants: CNAs (n = 6), a nurse, and a field program monitor for a non-governmental organization (NGO) implementing national community nutrition program activities. The key informants were included to add perspective and dimension to the data collected from the mothers and fathers. Because key informants regularly interacted with parents and lived within the community, they were able to provide valuable information regarding community perceptions and attitudes. This qualitative study is part of a larger research project and the fieldwork team already had established good rapport with the communities through an initial data collection process.

### Data collection

Guides for FGDs and interviews (Table 1) were developed based on previous relevant literature and the research questions. Questions were reviewed by the research team before translation in Malagasy. The questions in Malagasy were reviewed by native speakers: two mothers and two fathers of children younger than five years. No formal revisions were made to the questions before data collection. As data collection progressed, the researcher reflected on the responses given by the participants and the discussion after each FGD. As appropriate to the local context, the researcher revised the way the questions were framed based on the local context. The same approach was used for the interviews. FGDs and IDIs were conducted in the local language, Malagasy, by the lead author and were audio-recorded. On average, FGDs lasted for 40 minutes for mothers and 25 minutes for fathers. To achieve saturation, the researcher used probing questions during the interviews and FGDs. Saturation was reached when no new information was being shared during the FGDs or IDIs. At the time of data collection, the lead author, a native Malagasy from the capital city, was an educated male doctoral student.

**Table 1:** Guide for focus group discussions and interviews.

**Focus group discussion guide: mothers**
1. What kind of activities does your husband/partner do with the child? 2. What prevents him from being more involved in activities with the child? 3. How would you feel about getting him more involved in activities with the child? 4. How would you go about getting him more involved in activities with the child? 5. What would the neighbors/other community members think/say if he is more involved?
**Focus group discussion guide: fathers**
1. What are responsibilities of fathers regarding childcare? 2. What kind of activities do you do with your child? 3. How do you support your wife/partner in raising your children? 4. What activities would you like to do more with your child? 5. If you wanted to be more involved in child care, what prevents you from doing so? 6. What would the other fathers think if they saw another father being involved in child care activities more than usual?
**Interview guide: key informants**
1. What do people think are the responsibilities of the fathers regarding child care? 2. What prevents fathers from being more involved in activities with the child? 3. What would the neighbors/community members say if they see fathers getting more involved in activities with the child?

### Data analysis

FGDs and IDIs were transcribed verbatim by a native speaker for the project and these transcriptions were back-translated into English by the lead author. A thematic analysis approach was used to analyze the data [26,27]. First, the researcher familiarized himself with the data by actively reading the transcripts and the field notes. Codes were developed from patterns found in the data. Two teams of researchers coded the data using NVivo v. 12 (QSR International, Melbourne, Australia). The codes were cleaned by the researcher and reviewed by the team after the coding process. Codes with similar patterns were grouped into themes based on the research questions. Theme development was also conducted as a team. Discrepancies were discussed and resolved if researchers disagreed during coding or the development of themes. There was less than 5% initial disagreement during data analysis. Field notes were used throughout the data analysis and the writing process to add context to the data.

### Trustworthiness and reliability

To increase the trustworthiness of the results, triangulation strategies were used [28]. Information was gathered from different sources: mothers, fathers, and key informants. Triangulation was also used by collecting data from both group discussions and semi-structured interviews. Additionally, data were collected from participants from different communities in the rural areas of the Vakinankaratra region. Having multiple perspectives from participants living in different areas potentially increased the reliability of the data and helped to achieve saturation. Trustworthiness and reliability were further reinforced by the involvement of two other researchers during the development of codes and themes. Also, to minimize bias during the discussions, the researcher informed the participants that the study was independent of the Ministry of Public Health and any NGO working in the area.

### Ethics

This study was approved by the Oklahoma State University Institutional Review Board and by the Ethics Committee of the Ministry of Public Health of Madagascar. The purpose of the study was explained to eligible participants and they were given the opportunity to ask questions about their voluntary participation or the research project. Study participants signed informed consent before any data collection activity. During the FGDs and IDIs, the investigator confirmed that the participants were ensured of their anonymity and confidentiality of the information they shared. Participants were compensated for their time after the FGDs and the IDIs with iodized salt and cooking oil.

## 3. Results

Table 2 summarizes descriptive characteristics of the study participants. Fathers (mean 32 years) were older than mothers (mean 23 years). Most parents were smallholder farmers and more mothers than fathers had received secondary education. Fathers tended to listen to radio more frequently than mothers.

**Table 2:** Characteristics of the focus group discussions participants.

Variables	Mothers N = 46	Fathers N = 17
Age, mean	23	32
Household size, mean	5	4
Occupation		
Doesn’t work	2	0
Farmer	42	13
Other	2	4
Highest education level		
At least some primary	16	8
At least some secondary	19	4
Higher	8	5
Missing	3	-
ANC attendance		
Less than 4	12	-
4 or more	34	-
Radio listening		
Not listening	20	5
Once or twice a week	9	3
Almost every day	17	9

### Perceptions of fathers’ responsibility in child care

In all FGDs, fathers mentioned providing financial and material support for the household as their main responsibility (Table 3). Fathers perceived that their responsibility was limited to finding money for health care and buying clothes for their families. Also, fathers thought they were responsible for procuring the household’s material assets such as agricultural tools and other items in the house. Interviews with the key informants confirmed the fathers’ views.

“So, providing, looking for money” Father, FGD 2.

“Most of the time, men think that they should just provide” CNA, IDI 5.

**Table 3:** Themes for the perceptions of fathers’ responsibility in child care.

Themes	Data source	Times mentioned	Illustrative quotes
Financial and material support	Fathers FGD	12	“Yes, but even when the child is sick, you will need money so you have to be responsible. For foods and so on, for toys that the child may want because he will talk to the mother for that and she will send him to you” (Father, FGD 1)
	IDI	12	“Find money, they usually think about food, always food. And yes, that’s true that when they are leaving the house, they are looking for money so that’s a responsibility already. But when they found the money, they don’t think much about anything else. They would just give the money to the mother and ask her to get food so the wife goes and prepares the food” (Field Monitor, IDI 7)
General care activities and spending time when mothers are busy	Fathers FGD	13	“I do that sometimes [bathing] when my wife is busy” (Father, IDI 1) “The first responsibility a father has about child care is to soothe the child if the mother is not there, to feed him so that he can grow” (Father, FGD 2)
Support for mother with chores	Fathers FGD	8	“When raising a child, most of the time, we [fathers] have to get used to it so we have to participate in taking care of the child because the mother may be busy or you may even offer to do some of the things at home” (Father, FGD 3)
	IDI	1	“Also, he [the father] should be helping the mother when she is busy” (CNA, IDI 4)
No caregiving responsibilities for fathers	IDI	4	“The father rarely takes care of the child (pause). Even when preparing the food for the child, buying it from the market, everything about the child, the mother does it. I really don’t see what the fathers are doing” (Nurse, IDI 8) “Most of the time, men think that they should just provide” (CNA, IDI 5)
Child stimulation: playing and teaching	Fathers FGD	5	“You have to make sure his [the child] mind grows” (Father, FGD 2)
Console the child	Fathers FGD	5	“Making them happy when their mother is not there” (Father, FGD 3)
Supervise mothers	Fathers FGD	3	Supervising is then what the fathers do most of the time but the real work is for the mother only (Father, FGD 3)

FGD: Focus group discussions.

IDI: In-depth interviews with key informants.

CNA: Community nutrition agent.

Several fathers mentioned that they were responsible to help their wives in various child care activities such as bathing, cleaning, and feeding the child; but they thought they should help only when mothers were busy with other chores. Directly helping mothers with household chores also was perceived as fathers’ responsibility by a few fathers.

“For me when the mother is busy and if the baby is throwing up, then I go and clean him up” (Father, FGD 1).

“When you are home, you both have to fetch water because you both are tired. That should be normal if you love your wife. You wouldn’t stay there doing nothing because both of you are tired after work” (Father, FGD 3).

However, caregiving was not seen by the community as the fathers’ responsibility. The mother is the primary caregiver, but the father may help if he is willing and available.

“Yes, he [the father] does not take care of the child directly but when he has time, he can take the child and leave the woman to take care of the house” CNA, IDI 4.

Fathers also mentioned that they were responsible for teaching and playing with the child. Some fathers made the connection between play and development. Several fathers in all three FGDs mentioned supervising and advising as their roles in supporting mothers in raising their children. Supervision included ensuring that the mothers gave nutritious foods to the child and had appropriate hygiene practices. A few fathers in two FGDs mentioned consoling the child in the absence of the mother was an important responsibility of fathers.

### Fathers’ current involvement in child care activities

Child home stimulation, spending time with the child, feeding and occasionally preparing food for the child, and supporting the mothers with household chores were the reported activities related to child care in which fathers were involved (Table 4). Most fathers in the FGDs mentioned playing with and teaching their children regularly. Teaching included talking, walking, and even disciplining. Fathers also were engaged in various play activities with their children, from playing soccer to dancing with them. Also, some fathers were aware of the connection between playing and teaching and child development. Often, they reported the importance of helping the child’s mind to grow. Responses from the mothers were similar suggesting that playing and teaching the child were the main activities they saw their husbands doing with the children.

“They [fathers] teach the baby how to talk too” Mother, FGD 7.

“I teach him to say “dada” (father in Malagasy) or something like that” Father, FGD 2.

“They [father and child] play soccer” Mother, FGD 3.

“I play with him [child] to open his mind” Father, FGD 1.

**Table 4:** Themes and codes for the fathers’ current involvement in child care activities.

Themes	Codes	Data source	Times mentioned	Illustrative quotes
Child home stimulation	Teaching the child	Mothers FGD	9	“He [the father] talks to him, he shows him how to play” (Mother, FGD 2)
		Fathers FGD	7	“I show him things to develop his mind because little kids cannot stay at home for long” (Father, FGD 3)
	Playing with the child	Mothers FGD	37	“For example, playing soccer or playing homemade dolls or even making the baby dance on the bed” (Mother, FGD 4)
		Fathers FGD	8	“That’s how we all think about the child, so you raise him with love by playing with him” (Father, FGD 3)
General child care activities	Taking care of the child	Mothers FGD	6	“He [the father] changes his clothes” (Mother, FGD 6) “And when the baby is not well then taking care of him or taking to the health center” (Mother, FGD 4)
		Fathers FGD	5	“If they are dirty then you should take responsibility and clean them (Father, FGD 2)
	Spending time with the child	Mothers FGD	8	“They [fathers] can take a walk with the children, for example looking for things the children can play with” (Mother, FGD 7)
		Fathers FGD	2	“For me, I take him [the child] around” (Father, FGD 3)
	Holding the child	Mothers FGD	6	“When I am washing the clothes, I will make him hold the baby” (Mother, FGD 7)
		Fathers FGD	7	“For me too in the evening, I hold the baby, that’s what I do” (Father, FGD 1)
Food preparation and feeding	Cooking and feeding	Mothers FGD	10	“When the food is ready, they can feed the baby” (Mother, FGD 4)
		Fathers FGD	11	“She goes every Thursday to deliver potatoes to go to Antananarivo, so she doesn’t bring the children. Then I have to stay at home with my two boys. So, I prepare their foods and I feed them” (Father, FGD 3) “For example, when the mother is busy, I can prepare the food and feed him” (Father, IDI 1)
Support mothers	Help with household chores	Fathers FGD	10	“The way I help her, for me when it’s the evening like this, there are less things to do, so I go and get groceries and then I come back” (Father, FGD 1)
	Work on agriculture	Fathers FGD	1	“For example, if we plant, we do that together, maybe the women will plant or put the fertilizer, but we sow together, we plant greens together. Also, for example, when we weed the corn, when we have to dig, we do them together, some will put the fertilizer, some will sow” (Father, FGD 3)

FGD: Focus group discussions.

IDI: In-depth interviews with key informants.

CNA: Community nutrition agent.

Additionally, several fathers mentioned they frequently hold the child and walk around the neighborhood with the child, as well as cleaning the child on certain occasions.

“He [the father] takes him [the child] and they walk around, he shows him around” Mother, FGD 1.

Fathers also mentioned that they were involved in various child care activities such as changing clothes or washing the child’s hands. Mothers confirmed in the FGDs that fathers participated in these activities when the mothers were busy.

“[My husband] washes the baby’s hands when dirty” Mother, FGD 2.

Some fathers reported being involved in cooking and feeding the child as well, but only when mothers were not available. Discussions with mothers confirmed that fathers cooked and fed the child sometimes when they were busy. Also, fathers were still partly relying on mothers when they participated in such activities.

“For me, it’s very similar, if I am home then the mother will show me what foods the baby should be eating so I learn from her and when she is not there I do it” Father, FGD 2.

Only a few fathers mentioned helping mothers do household chores and agriculture work as part of their support in raising the child.

From the FGDs, a few fathers expressed interest in getting more involved in child care activities, but only when they are not busy. One father mentioned that participating more with tasks related to his child will build stronger bonds. Almost all mothers in the FGD were enthusiastic about increasing fathers’ involvement in child care activities. Mothers stated that the child would be healthier and stronger when fathers were participating more in child care activities. Moreover, mothers reported they would be relieved, happy, and would have fewer chores as well as more time to do other chores.

“For me, if I have time, just as he said earlier because of work. But if I have time and I am home, then I must take care of the child. But when I am away, then the mother will do what she can” Father, FGD 1.

“If he [the father] is more involved, the baby will be healthier and stronger because even his [the child] food will be appropriate” Mother, FGD 1.

“Because if he [the father] holds the baby, we can do all of those things…we could wash clothes, sweep the yard, talk more to the baby, if he is there holding the baby or if he participates more” Mother, FGD 4.

Only a few fathers mentioned activities they were interested in doing more with their children, including playing and walking around more. Mothers also wanted fathers to be more involved in activities such as playing with the child, holding the child more, and participating more in feeding. Several mothers wanted fathers to help more with household chores and agriculture work.

“I would like to make him [the child] some toys if I had time because they like those things, and it makes their mind grow” Father, FGD 2.

“I would like to take my child around but I don’t have time. I would like to take her around the fields but there is no time so I can’t do it” Father, FGD 1.

“I would ask him [the father] to prepare the foods for the baby and feed the baby after” Mother, FGD 7.

“I would ask him to help around the house” Mother, FGD 7.

### Barriers to fathers’ involvement

Work and related factors, gender role stereotypes, lack of willingness, and not being prepared were identified barriers to fathers’ involvement in child care (Table 5). Both fathers and mothers in their FGDs stated that fathers had limited time to take care of the child because of their work and their schedule. Also, men engage in casual labor in agriculture, and they work for longer periods than women, usually the whole day. Thus, even those who are willing will only have time in the evening and the weekends to interact with the child and give support to the mother.

“He is the one who looks for money so he doesn’t have time” Mother, FGD 6.

“The main problem is, as mentioned earlier, our schedule, our work schedule” Father, FGD 3.

**Table 5:** Themes and codes for the barriers to fathers’ involvement in child care activities.

Themes	Codes	Data source	Times mentioned	Illustrative quotes
Fathers’ work and related factors	Limited time because of work	Mothers FGD	23	“He [the father] is busy working all the time so he can’t take care of the baby” (Mother, FGD 1)
	Limited time and unavailable because of work	Fathers FGD	11	“The main problem as discussed earlier, the main problem for us is that we are not always home even if we wanted to be more involved” (Father, FGD 2)
	Separation and distance	Fathers FGD Mothers FGD	9	“Sometimes we stay there for weeks or even for months, depending on the contract. Some are as long as 5 months” (Father, FGD 2) “For example, when he is working in Ambatondrazaka or Maevatanana (bigger towns in another region), he is gone for months, one or more, so he can’t be with the baby” (Mother, FGD 2)
	Tired from work	Mothers FGD	8	“They [fathers] are tired from work and they don’t want to do anything anymore” (Mother, FGD 7)
		Fathers FGD	2	“For me when I am tired from the fields” (Father, FGD 1)
		IDI	12	“When the fathers are tired from work, they don’t care about their children” (CNA, IDI 2)
Traditional gender roles in child care	Mothers are the primary caregiver	Mothers FGD	16	“He [the father] may do things when you [the mother] are not around. He will take care of the baby if you are not around for half a day or so. But if both of us are there and if the child is vomiting, he will ask me to take care of it” (Mother, FGD 1)
		IDI	6	“And some [fathers] would say that the women are there for taking care of the children” (Nurse, IDI 8)
	Fathers are providers, not caregivers	Fathers FGD	1	“For us men we have to provide so we don’t have to be so close to the child, not more than their mothers” (Father, FGD 1)
Lack of willingness from the fathers		Mothers FGD	8	“He [father] even says sometimes that I don’t know how to take care of babies…but when I am not around he knows how to take care of them but it’s only when I am around that he doesn’t want to do it” (Mother, FGD 4)
		IDI	7	“It’s the willingness that is lacking, they just don’t want to” (Field Monitor, IDI 7)
Fathers not prepared		Mothers FGD	17	“They [fathers] can’t understand all of the things you have to do to take care of a child. For them, when they are clothed and fed then that’s it. They don’t know how to take care of the other things” (Mother, FGD 3)
		IDI	5	“It’s just not their habit, they are not used to do it. They haven’t been doing it because the women have always been doing it” (CNA, IDI 5)
Fathers are irritable and worried		Mothers FGD	3	“Sometimes they [fathers] are irritable” (Mother, FGD 3) “Also, sometimes, there is something that worries him [father] so he won’t touch the baby” (Mother, FGD 3)
		Fathers FGD	1	“And if there is something else that worries me, then it is hard to take the baby so I go away for a bit” (Father, FGD 1)

FGD: Focus group discussions.

IDI: In-depth interviews with key informants.

CNA: Community nutrition agent.

In a few communities, several men work in larger cities or in locations where there is a high demand for a physical workforce. Men leave their homes for long periods of time, ranging from 2 weeks to 9 months, to work because the pay is better than what is offered in rural areas.

“The first thing is time because some children are even scared of us when we come back home. They are scared because they don’t recognize us” Father, FGD 2.

Also, fathers reported being tired from work in the evening so they don’t want to take care of the child or to be involved with additional child care activities, which was confirmed by several mothers in the FGDs. Almost all of the key informants also stated that fathers being tired from work is one of the main barriers to their involvement.

“They are tired from work, from looking for money, so they are very tired and cannot take care of the child [in addition]” CNA, IDI 6.

Traditional gender roles regarding child care were also identified as barriers to fathers’ involvement. Mothers mentioned that caregiving is seen as the mothers’ job, and the father may take care of the child only when they are not available. Several key informants confirmed these perceptions by saying that women were expected to take care of the child. In line with the mothers’ and the key informants’ responses, fathers stated that they are providing already and should not be involved much in caregiving activities, which are women’s responsibilities.

“You are the mother so you have to take care of the baby” Mother, FGD 1.

“So, there are fathers who are really saying that the women are the ones who take care of the children” CNA, IDI 2.

“Those [child care activities] are women’s job but the men just advise” Father, FGD 3.

Several mothers in a majority of the FGDs commented that fathers were not willing to be involved in child care activities. The fathers’ laziness was also mentioned to be a barrier to involvement. Some mothers reported that fathers will not do anything even if they were asked. According to a few mothers, some fathers would pretend not to know how, but they will take care of the child when the mothers were not available. Several key informants also provided similar responses that some fathers are not willing to be involved in child care activities. Interestingly, nothing similar was discussed in the fathers’ FGDs.

“No, they [fathers] don’t say anything but whenever you ask him [the father] to feed the baby, he will just say no” Mother, FGD 4.

“I think they [fathers] know but they just won’t do it” CNA, IDI 3.

However, several mothers also believed that their husbands were unprepared to take care of the child. Several mothers mentioned that some fathers do not know how to take care of infants or how to perform specific activities such as cooking or feeding. The key informants commented that taking care of infants is not in the fathers’ habits, so they don’t do it.

“For mine, it’s because he doesn’t know how to prepare food that he doesn’t do it” Mother, FGD 3.

A few mothers reported that fathers are not involved in child care because they are irritable, especially after work so they do not want to be bothered, and some are worried. A father in the FGD mentioned that when he is worried, he did not want to be near his child.

### Community perceptions of involved fathers and their wives

Three themes were extracted including traditional gender roles regarding child care, perceptions of involved fathers, and attitude towards the wives of engaged fathers (Table 6). Similar to the parental perception of fathers’ responsibility and the barriers to involvement, the community considers caregiving as the mothers’ job.

“[They will say] That’s [caregiving] for the women” Mother, FGD 5.

“So, the people are saying that those [child care activities] are women’s work, and if a man does those, they would start gossiping about it” Nurse, IDI 8.

**Table 6:** Themes and codes for the community perceptions of involved fathers and their wives.

Themes	Codes	Data source	Times mentioned	Illustrative quotes
Traditional gender roles on child care: caregiving is mothers’ job		Mothers FGD	9	“They [other fathers] will say that those are the women’s responsibility, not theirs” (Mother, FGD 3)
		Fathers FGD	4	“They [the community] will ask if there is no woman in the house that you would have to do that [taking care of the child]” (Father, FGD 1)
		IDI	6	“Some [people] would just say that why would they care about that, that the father should not do all of those things [child care activities], they would ask what does the mother do” (CNA, IDI 2)
Perceptions of involved fathers	Involved fathers are controlled by their wives	Mothers FGD	6	“Because they are men, they won’t let women undermine them. His friends will ask him why does he wash the clothes when the wife is there” (Mother, FGD 4) “Your wife is controlling you, she makes you do chores”, that’s what they would say” (Mother, FGD 6)
		Fathers FGD	1	“They would say that the man doesn’t stand for himself, the woman has overpowered him, etc.” (Father, IDI 1)
		IDI	2	“They would say that the man is being controlled by the wife, that the wife has him under her control, and that he is doing whatever she says” (CNA, IDI 2)
	Involved men are weak and fools	Mothers FGD	4	“That he [the father] is defeated by his wife” Mother, FGD 7
	Involved fathers are discouraged by the community	Mothers FGD	3	“Nobody will ever encourage a man to wash clothes (laughter)” (Mother, FGD 4)
	Involved men are good fathers	Mothers FGD	16	“They will say: “Look at this man, he is doing this and that, he really takes care of his child, he is taking his responsibilities towards the child, not like you [her own husband]” (Mother, FGD 5)
		Fathers FGD	5	“For me, I think the mothers will encourage because they love their children, it’s in them, men can love their children but their mothers carried them for 9 months so whenever they see other people’s children being taken good care of, they would say that those children are lucky because their dad is very responsible doing those things” (Father, FGD 1)
		IDI	5	“They would be happy, they would say that the father really loves his children, they would be happy” (CNA, IDI 3)
	Involved fathers are lazy and laughable	IDI	2	“They would say that, that man is lazy, which happens a lot actually. They would say that man doesn’t want to work so he is just taking care of the child” (CNA, IDI 5)
		Mothers FGD	1	“Men don’t wash clothes. You can’t see men grinding paddy, they will be embarrassed even if they want to do things because the other people will make fun of them so they won’t do it. That’s most of the men in the countryside” (Mother, FGD 4)
Attitudes towards wives of involved fathers	Wives of involved fathers are not good mothers and wives	Mothers FGD	18	“Yes, lazy. They will say that she is not like other people’s wives, they can do it all but not her, she has to rely on the husband” (Mother, FGD 5)
		Fathers FGD	1	“They will say that my wife is not doing anything at home because I am doing all the work” (Father, FGD 1)
		IDI	1	“The wife [of the involved father] is not polite and she doesn’t know what she is supposed to do. Some mothers can say that” (CNA, IDI 3)
	Neighbors are envious of the wives of engaged men	Mothers FGD	18	“They would envy the wife [of the involved father]: “If only my husband was like that” (Mother, FGD 7)
		IDI	2	“They would envy her [wife of the involved father], they would tell each other their problems and say if only their husbands were more like that” (CNA, IDI 5)

FGD: Focus group discussions.

IDI: In-depth interviews with key informants.

CNA: Community nutrition agent.

Several mothers and fathers in the FGDs mentioned that such comments come mostly from other fathers. If men participate in child care activities or help their wives with household chores, other fathers and members of the community will tease them. Men engaged in child care activities are usually seen as weak or fools or submissive to their wives. Mothers and the key informants reported that other men are the members of the community who are the most critical towards involved fathers.

“They [other fathers] would say that he is a weak man” (Father, FGD 3).

Several mothers commented that the community would discourage engaged fathers, even if they wanted to participate more. Involved fathers would also be considered lazy and would be ridiculed. Because men are expected to provide, they would be considered lazy if they are taking care of the child instead of working hard in the fields.

“They [the neighbors] would discourage him” Mother, FGD 6.

However, several mothers in the majority of the FGDs and a few fathers stated that other mothers encouraged involved fathers if they saw them participating in child care within the community. Fathers actively engaged in child care were seen by other mothers as responsible and loving. Most of the key informants also confirmed the encouragement from other mothers.

“They would say: “this man is really responsible, he takes good care of their baby, he can do it all” Mother, FGD 1.

“They may say: “you are a wise man and you love your children, you wash clothes and all” Father, FGD 3.

In the majority of the FGDs, several mothers reported that wives of involved fathers were not considered good mothers. They are often seen as lazy, rude, or unable to properly take care of their children based on the gender roles associated with child care. However, in almost all of the FGDs, mothers mentioned that they would be envious of the wives of involved fathers. Mothers consider them lucky to have a supportive husband and that they work well as a team in raising their children.

“You are really lucky with such a husband!” Mother, FGD 5.

## 4. Discussion

Results showed strong gender role differences in child care among Malagasy parents; mothers and fathers have distinct roles. The gender role expectations were a salient theme across the data and influenced community perceptions regarding involved fathers and were identified as a barrier to their involvement. Fathers primarily see themselves as providers, not caretakers, a perception widely held by the larger community as well. Other qualitative studies in LIMCs also reported similar findings where men’s responsibility was limited to providing financial support for their families [6–8,29,30]. But fathers were also expected to interact with the child by teaching and playing with them. Mothers, on the other hand, were seen as the primary caregivers for the children, responsible for feeding and hygiene. However, because most of the households had low income, mothers were also providing for the family by working as casual laborers. Thus, mothers appeared to have a desire that household chores and child care activities are shared within the household. During the focus group discussions, they mentioned wanting fathers to be more involved with such activities. Also, they expressed envy towards mothers whose husbands were more engaged in daily activities around the house.

Compared to other studies from African countries [6,7,30], fathers in our study seemed to be more involved in child care, but their involvement depended on the activity and the availability of the mothers. Fathers reportedly took part in feeding, cooking, or cleaning the child only when the mothers were unavailable, probably because of the perception that these tasks were the mothers’ primary responsibility. Most of the fathers’ time with children was spent on companionship and play. Similar results were reported in different settings as European, American, and Indian fathers engaged more in physical and stimulating play than mothers [31,32]. Father-child play is particularly important for the child’s language skills and social-emotional development. Fathers’ playfulness has been associated with higher receptive language scores especially during play pretend activities [18]. Compared to mothers, fathers may use more words and more elaborate grammar as well as full sentences [32,33]. Also, the rough-and-tumble play between father and child was associated with socioemotional competence and self-regulation in a meta-analysis [34]. Malagasy fathers may need to be encouraged to talk to and play with their children regularly in addition to just holding them to actively promote optimal child development.

At the household level, fathers’ work schedules restricted their interaction with their children as some of them worked all day or even away from home for extended periods of time. That household-level barrier was mentioned in all FGDs and in most of the key informant interviews and therefore highlight its importance in fathers’ involvement. The socioeconomic context of the community may play a key role as most households practice subsistence agriculture and experience frequent food insecurity. With most fathers being casual labor workers paid hourly, they prefer long hours of work to increase their wages. Also, the opportunity to work in different towns or cites is appealing for fathers with more skills, such as carpenters or construction workers as the salary is generally higher. Thus, fathers would sacrifice their father-child interaction to provide for their families as it is what they perceive as their first responsibility.

At the community level, gender role stereotypes in child care were barriers to fathers’ involvement. Consequently, fathers participating in child care activities risk being ridiculed by the community and their wives would be criticized as irresponsible mothers unable to take care of their children. Community perceptions are important because they influence individual behaviors and actions [35]. For example, the negative attitude towards the wives of involved fathers may reinforce maternal gatekeeping; the community criticism of their maternal capabilities ultimately limits the husbands’ involvement. Changing these social norms could encourage fathers to be more engaged if involvement is perceived positively.

Although there is accumulating evidence of the positive results of fathers’ involvement in other areas than financial support on maternal and child outcomes, challenging these perceptions may be difficult as they may be deeply rooted in the culture. Increasing paternal involvement may require changing the traditional perceptions around the distinct roles of parents in child care activities. Raising awareness of the benefits and the importance of the engagement of both parents in all aspects of child care may be needed. Both parents contribute uniquely to child development because of the differences in the dynamics of their relationships with the child. Having direct interactions and activities with involved fathers and mothers, rather than one parent alone, may expose the child to a wider variety of stimuli that will likely improve developmental outcomes [16]. Additionally, involving fathers in activities regarding child care, or even broadly in unpaid work, may alleviate women’s workloads. In our study, mothers also were providing for their families like fathers, in addition to the care of children for which they are primarily responsible. As mentioned by the mothers during the focus groups, having their husbands’ help will allow them to have more time to interact with their children.

There were few disagreements regarding fathers’ involvement between FGDs with fathers and mothers. Only fathers mentioned they helped their wives with household chores and other agricultural activities. Either fathers in the FGDs wanted to appear more involved in dealing with chores than they really are, or mothers did not feel their husbands were helpful. Also, parents had different activities in mind when asked about how fathers can be more involved. Fathers wanted to do more of what they were already doing with their children such as playing and walking around. Mothers, in addition, wanted their husbands to do more chores around the house. Different expectations on the responsibilities of the fathers may explain these disagreements. Wives’ perceptions and attitude towards their partners’ involvement have been shown to influence the level of engagement of fathers [36,37]. Men are more likely to be involved when they are encouraged and supported by their wives in engaging in child care activities. However, in conservative societies such as rural Madagascar and as evidenced by the responses during the FGDs, it is less probable that mothers will encourage openly their husbands to engage in child care activities as long as they are available. Additionally, although not explored in this study, Malagasy mothers may have some gatekeeping characteristics where they, consciously or unconsciously, prevent fathers from being more involved. Maternal gatekeeping is deeply rooted in maternal identity and has been documented in middle- and high-income countries [36,38–40]. In this study, mothers reported that their partners were unprepared and unequipped to take care of their children, a possible aspect of maternal gatekeeping. Mothers believing that fathers, unlike them, are unable to be suitable caregivers may restrict men’s involvement in child care. Not receiving positive feedback and encouragement, fathers may feel incompetent and will be less likely to be involved [41].

There are opportunities to increase fathers’ involvement in the Vakinankaratra region. Several fathers in this study mentioned that men’s responsibility goes beyond provision and that they should also help their wives with household chores when their wives are busy. This finding suggests that some fathers are interested and willing to participate in other aspects of child care in addition to their perceived responsibilities for financial support and child play. Fathers identified the potential benefits of stimulating activities (playing and teaching) on their children’s development.

Based on the findings, a few suggestions can be made to increase fathers’ involvement in child care activities in the Vakinankaratra region. First, increasing awareness of the community about the importance of fathers’ involvement in child health and family life in general, using culturally sensitive messages, may change the community’s perception of engaged fathers and facilitate their participation. Such strategies may be more effective using theory-driven interventions that address each of the attitudes and beliefs fathers and mothers in the community had towards involved fathers and their wives. A parenting intervention in Uganda with a component of family caregiving successfully changed the local attitudes on fathers’ involvement [42]. Parents understood the importance of involving fathers in other aspects of child care, especially in learning and development. Second, encouraging fathers to continue their current involvement may be important as well and may facilitate their participation in other activities in childcare. Mothers play an important role in encouraging their husbands to be more involved as maternal attitudes and beliefs of fathers’ roles in child care have been shown to be more predictive of fathers’ involvement than the fathers’ perceptions [36,43,44]. For fathers working away from home and lacking time, increasing parents’ communication on how men can still engage in child care may facilitate their direct involvement. Finally, gradually encouraging fathers to participate in those activities in which they expressed interest may be easier than drastically changing the responsibilities in the household. Trainings on detailed activities in which fathers can regularly engage may also be helpful. In the context of the rural Vakinankaratra region, such interventions may consider incorporating local income-generating activities as fathers’ work was, in addition to gender role stereotypes, a main barrier to fathers’ involvement.

The selection of the fathers is a limitation of this study as due to their work, only about a third as many fathers as mothers were available and willing to participate in the FGDs. The fathers who participated in the study may have been those more involved in child care activities. Fathers in our sample also had less education than most men in Madagascar. Despite these limitations, this study is the first to explore fathers’ involvement in child care and their potential in promoting nutrition and developmental outcomes in the highlands of Madagascar. Furthermore, several complementary data collection methods were used: FGDs with mothers, FGDs with fathers, and IDIs with key informants. Also, data were collected from 12 different communities in the Vakinankaratra region to increase the reliability of the results.

## 5. Conclusion

In the Vakinankaratra region, traditional gender roles in child care influenced the community’s perception of fathers’ responsibility and were one of the barriers to their involvement. Fathers are seen as providers and mothers are considered as caregivers. When alone with their children, fathers play and teach them, or hold them when the mothers are busy. Both mothers and most of the fathers were interested in engaging fathers more in child care but another barrier was the fathers’ lack of time or physical separation due to work. Interventions targeting both mothers and fathers and the community at large to change perceptions and attitudes towards involved fathers and their wives as well as the importance of shared child care responsibilities may increase fathers’ involvement in child care in the region.
